# An Efficient ERP-Based Brain-Computer Interface Using Random Set Presentation and Face Familiarity

**DOI:** 10.1371/journal.pone.0111157

**Published:** 2014-11-10

**Authors:** Seul-Ki Yeom, Siamac Fazli, Klaus-Robert Müller, Seong-Whan Lee

**Affiliations:** 1 Department of Brain and Cognitive Engineering, Korea University, Seoul, Republic of Korea; 2 Machine Learning Group, Berlin Institute of Technology, Berlin, Germany; UCLA, United States of America

## Abstract

Event-related potential (ERP)-based P300 spellers are commonly used in the field of brain-computer interfaces as an alternative channel of communication for people with severe neuro-muscular diseases. This study introduces a novel P300 based brain-computer interface (BCI) stimulus paradigm using a random set presentation pattern and exploiting the effects of face familiarity. The effect of face familiarity is widely studied in the cognitive neurosciences and has recently been addressed for the purpose of BCI. In this study we compare P300-based BCI performances of a conventional row-column (RC)-based paradigm with our approach that combines a random set presentation paradigm with (non-) self-face stimuli. Our experimental results indicate stronger deflections of the ERPs in response to face stimuli, which are further enhanced when using the self-face images, and thereby improving P300-based spelling performance. This lead to a significant reduction of stimulus sequences required for correct character classification. These findings demonstrate a promising new approach for improving the speed and thus fluency of BCI-enhanced communication with the widely used P300-based BCI setup.

## Introduction

Brain-computer interface (BCI) systems provide a direct electronic interface to translate messages and commands from the brain of the user to external devices without muscular control. To date, a major part of BCI research is focused on the restoration of communication [1]–[3]. Therefore, BCI's have been particularly utilized for patients whose motor and communicative abilities have been impaired by severe neuromuscular diseases such as amyotrophic lateral sclerosis (ALS). Those affected suffer from a gradual loss of voluntary muscular control due to motor neuron degeneration [4]–[6].

The P300 is the ERP component with the strongest deflection and has been widely investigated over the past few years. P300-based BCIs constitute arguably the largest category of BCI research. The first P300-based matrix speller (aka P300 Speller) was introduced by Farwell and Donchin [7]. Since its introduction it has been studied extensively by many research groups. In this conventional paradigm, a letter-matrix consisting of the alphabet and digits, arranged in a 6
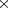
6 grid, is displayed on a computer screen and presented to the subject. While the subject attends to the specific letter they wish to spell, the rows and columns are flashed consecutively in a random order (the so-called classical Row-Column (RC) paradigm). When a row or column is flashed, that contains the attended letter, an elevated P300 can be detected in the subjects' EEG.

Since the publication of this paper in 1988 many extensions to the original RC paradigm have been proposed in order to improve its performance in terms of speed and accuracy (see [8] for a recent review). Some of the various configurations include: (1) electrode montages [9], (2) stimulus (or matrix) property alteration (i.e. color, size, rate and motion) [10]–[16], (3) variations of inter-stimulus intervals (ISIs) (or stimulus onset asynchrony (SOA)) and target-to-target intervals (TTIs) [10], [17]–[19], (4) various pattern recognition/machine learning algorithms for feature extraction [20]–[22], and classification [9], [23]–[25].

Furthermore, other groups have made a constant effort on the redesign of novel visual stimulus representation patterns for improving the P300 speller. Guger *et al.* [26] propose a single character (SC) speller, where characters are flashed individually in a randomized order. They compare the SC speller with the classical RC speller for 38 subjects and demonstrate that although the SC paradigm produces larger P300 responses than the RC paradigm, the RC paradigm still retains a higher performance than their novel SC paradigm. They attribute their findings to the increased fatigue as a result of the longer sequence of character selection. Two other research groups (Fazel-Rezai and Abhari [27] and Treder *et al.* [28]) propose a P300 speller, where the desired letter is chosen, based on a two-step process. Letters are grouped and randomly flashed, as opposed to the row and column intensification. The subject needs to identify and select the group containing the desired letter in the first step. During the second step the desired character is selected within that group. Treder *et al.* [29] further extends this two-step process by 3 alternatives (they are termed, "Hex-o-spell", "Cake-", and "Center- speller") using covert spatial attention and non-spatial feature attention modalities. Recently a series of papers has also considered online adaptation and unsupervised learning for BCI ERP spellers (see [30]–[32]).

Townsend *et al.* [33], (see also [34]) investigated a checkerboard paradigm (CBP) to overcome the following two issues: 1) adjacency-distraction errors which can occur when neighboring items flash with respect to target items and 2) double-flash errors, which occur when the same character flashes sequentially. The original CBP presents stimuli in an 8
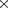
9 matrix and then separates the letters into 2 groups (a white and a black group each in a 6
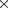
6 matrix). By disassociating the rows and columns, the CBP can overcome 'repetition blindness' [35] by introducing the constraint that a minimum of six intervening flashes (of non-targets) should be between targets and the 'flanker effect' [36] by only simultaneously flashing letters which are not in the same row or column.

Finally, Jin *et al.* [37], [38] designed a novel stimulus presentation pattern that requires fewer flashes than RC and SC paradigms. They test a number of different flash patterns as well as adaptively detecting the necessary number of flashes to average. Their findings indicate that they are able to reduce the numbers of flashes, as well as minimizing the interference from items adjacent to targets.

In recent years, a number of groups have focused on changing the stimuli from intensified characters to alternative stimuli such as faces. In particular, face stimuli based approaches elicit not only P300 responses, but also face-specific ERP components, namely N170 and N400f. N170, a negative deflection at around 140-200 ms after the onset of the stimulus presentation, is known to show a stronger deflection when faces are presented as compared to other stimuli [39]. When compared with unfamiliar faces, familiar faces elicit an enhanced negativity between 300 and 500 ms ('N400f').

Based on the above-mentioned face-specific temporal features, Kaufmann *et al.* [40] adopted famous face images and superimposed them with the letters of a P300 matrix speller. In their study, the face-sensitive ERPs show an enhanced accuracy due to the contribution of the N170 and N400f features, which are accompanied by the recognition of familiar faces. In addition, they could show in [41] that face stimuli can be helpful to avoid BCI inefficiency [42] for patients with neurodegenerative diseases. Zhang *et al.* [43] also utilized stimuli based on configural processing of human faces in an oddball paradigm. Also here, face configuration related ERP components such as N170 and vertex positive potentials (VPP) result in higher accuracies, as compared to the conventional P300-based BCI with stimuli of intensification patterns. A number of previous studies have investigated, whether face emotion has an effect on BCI performance, however to date no performance differences have been found for these type of stimuli [41], [44].

In this paper, an offline study is performed, where two improvements to the above mentioned issues of P300 spelling are examined: 1) to minimize adjacency-distraction errors we adopted a random set-based stimulus representation pattern (RASP), similar to a previous study [33]. However, in this previous work, two factors were manipulated: Not only did they alter the (random) groups of letters flashed simultaneously, but also tried to minimize the double-flash related problems by ensuring a minimum of six intervening flashes between targets. As a result, it was not possible to determine, which of these factors were responsible for the increased performance and to which extent. In this study we did not define a static TTI and this enabled us to examine the effect of various TTIs. By isolating the two factors (i.e. the required minimum TTI as well as the random set-based stimulus representation pattern) it is now possible to more accurately quantify the benefit of the two individual approaches. 2) effects of face familiarity on P300-based BCIs: The present offline study is dedicated to further investigate the effects of face familiarity on the performance of BCIs using stimuli of facial images. In a previous study, we found that brain activity responses to one's own face are markedly unique and show stronger responses when compared to familiar or unfamiliar non-self faces and this phenomenon was defined as 'face-specific visual self-representation' in [45] for the neurophysiological basis thereof we refer to [46]. These results were obtained in a previous person authentication study [47]. Earlier studies have also shown that task complexity shows a strong positive correlation with the amplitude of the ERP responses [48], however habituation effects, which may be caused by repeated presentation of the same stimulus, could counteract this effect [10]. To this end we designed the paradigm, such that self-faces as well as non-self faces are presented in a randomized order.

## Materials and Methods

### Participants

Fifteen healthy university students who were between 26 and 32 years (mean 27.7
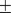
1.5, right-handed, all males) took part in our experiments. All participants had normal or corrected-to-normal vision. None of the participants had a previous history of psychiatric, neurological, or other diseases that might otherwise affect the experimental results. All experiments were conducted according to the principles expressed in the Declaration of Helsinki. This study was reviewed and approved by the Institutional Review Board at Korea University and written informed consent was obtained from all participants before the experiments. Participants were seated comfortably in a chair with armrests in a quiet room at a distance of 60
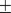
5 cm from a standard 19 inch LCD monitor (60 Hz refresh rate, 1280
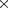
1024 screen resolution) which corresponds to an angle range from −10°
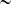
10°. During the experiment, they were asked to relax while remaining attentive and avoiding unnecessary movement.

### Equipment and data acquisition

**Figure 1:**
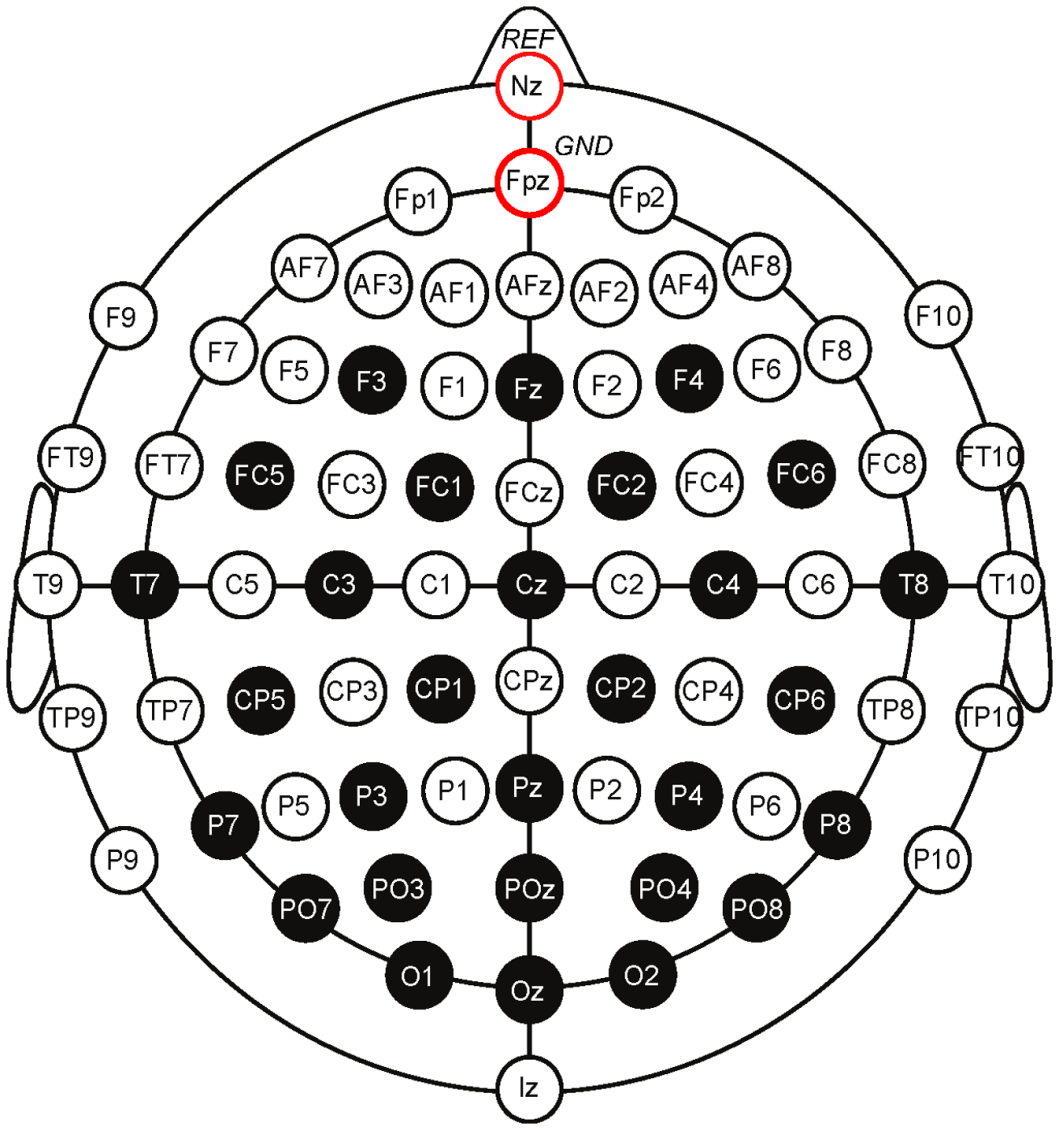
The selected electrode locations of the International 10–20 system (29 EEG recording electrodes (black circles), one ground and one reference electrode (red circles) used in this paper).

EEG signals were recorded with a sampling rate of 500 Hz with a BrainAmp multichannel EEG amplifier by Brain Products from the following 29 Ag/AgCl electrodes on a cap (actiCAP, Brain Products, Munich, Germany), according to the international 10–20 system: F3, F4, Fz, FC1, FC2, FC5, FC6, C3, C4, Cz, T7, T8, CP1, CP2, CP5, CP6, P3, P4, Pz, P7, P8, PO3, PO4, POz, PO7, PO8, O1, Oz, O2 (see Figure 1). Channels were nasion-referenced and grounded to electrode Fpz. EEG signals were then down-sampled to 100 Hz with a 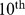 order digital Chebyshev filter. The impedances of the EEG electrodes were below 10 k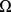. EEG data was amplified and digitized using BrainAmp hardware (Brain Products, Munich, Germany).

**Figure 2:**
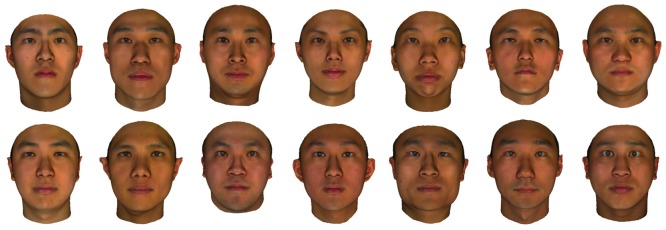
Examples of the face stimuli obtained by the 3dMD face capture system with the same illumination conditions. One self-face image and a number of non-self-face images are depicted here.

For acquiring face images a 3dMD face capture system was used ensuring the same lighting conditions for all subjects (http://3dmd.com) making a neutral facial expression, while facing the camera. Face images were derived from front-view photographs using Adobe PhotoShop software. All the face images were processed to remove external features such as hair and then cropped into a common oval frame which was placed on a black uniform background. Face images were scaled to an image size of 400
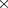
500 pixels. These final face stimuli were presented as in Figure 2 (for further details, please refer to [47]).

### Experimental stimuli and paradigm

Three different spellers, all derived from the original P300 speller, were examined. Each speller allowed the user to choose one out of 36 unique symbols, comprising the letters of the English alphabet (A to Z), digits (1 to 9) and underbar (_). All three matrix spellers were presented with a 6
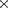
6 matrix and highlighted characters or faces were flashed consecutively in random order. The three spellers were implemented with the Psychophysics Toolbox (http://psychtoolbox.org). The Row-Column (RC) condition corresponded to the traditional approach [7]. In the RC condition each sequence necessary to select a target (i.e. letter to spell) comprised 12 stimulus flashes of each row and each column.

In the first proposed variant, called random set-based stimulus representation pattern (RASP), letters were randomly shuffled in a virtual six-by-six matrix, prior to stimulus presentation and then 12 stimulus flashes were presented to the subject. As a result users saw a unique combination of letters in each stimulus during a given sequence. The number of stimuli were equal for the RC and RASP paradigms and the temporal distribution of TTIs was the same on average. Similar to the RC condition, each letter was flashed twice within a sequence. In other words, in a series of 12 flashes, the target letter (but also every other letter) was contained in two of the twelve flashes.

**Figure 3:**
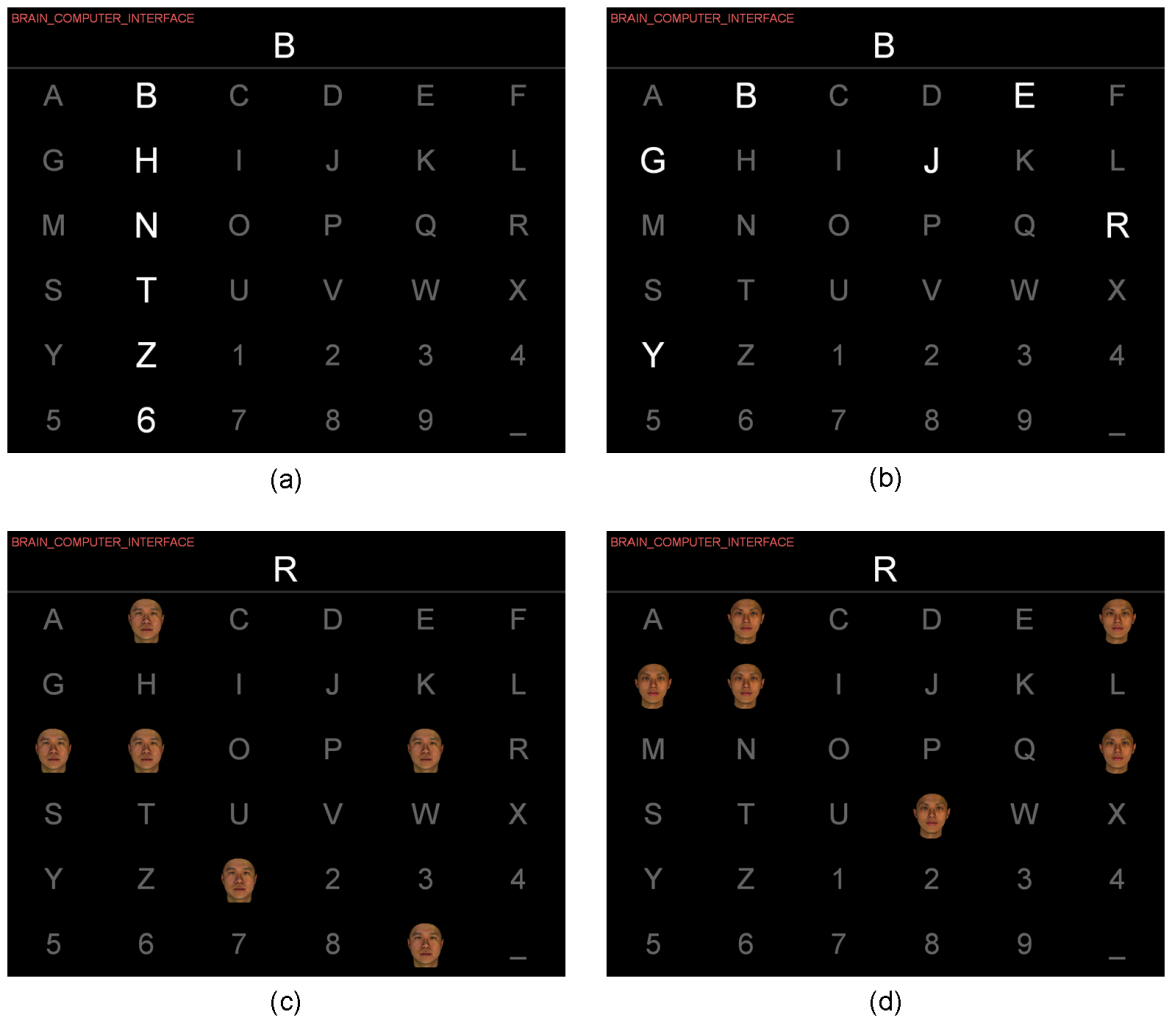
The different conditions of the paradigm. (a) The classical row-column (RC) paradigm, (b) The proposed random set presentation (RASP) paradigm, (c) RASP paradigm with flashing self-face in one row of the virtual matrix, (d) RASP paradigm with flashing non-self-face in one column of the virtual matrix. Both RASP and RASP-F stimuli were shown semi-transparently to the participants such that the characters were still visible. However, this is not shown here for illustration purposes.

As a second variant, also based on RASP, the characters were overlaid with face stimuli. This variant was termed RASP-F. The face images were semi-transparent to allow for uninterrupted focusing on the target letter while the face stimuli were flashed. The types of face stimuli, which were used in the experiments can be divided into 2 categories. Self-face and non-self-face images were used for stimulation. A self-face image consisted of the image of the subject, while a non-self-face image consisted of a familiar face such as his/her friends or of unfamiliar faces whom he/she has never seen before. In the case of self-face presentation, the same face image was presented as a stimulus but in the case of non-self-faces different face images were presented each time in order to counter the effect of habituation. When a row was selected in the virtual 6 by 6 matrix, the letters contained in this row were flashed with self-faces in the speller. Similarly, when a column was selected, contained letters were flashed with non-self-faces. Therefore, the ratio between self-face and non-self-face presentation was 50∶50. See Figure 3.

During the experiment, participants were instructed to sit still, relax their muscles and try to minimize eye movements. Each experiment consisted of 2 phases: a training phase and a test phase. Training and test phases were recorded on two separate days. Transfering classifiers from one session to another is known as *session-to-session* transfer and known to lead to (slightly) reduced classification rates. The presentation order of the spellers was randomized across participants. In each session, participants were provided with strings of letters they were supposed to spell. The whole string was displayed at the top left of the monitor and the next item-to-spell (the target letter) was displayed above the letter matrix (see Figure 3). During the initial training phase, subjects had to copy-spell one sentence 'BRAIN_COMPUTER_INTERFACE'. There was no feedback and EEG was recorded for offline analysis. In the second phase subjects had to copy-spell another sentence 'KOREA UNIVERSITY' (without the space). The participant's task was to attend to (or count) the number of times the target character flashed. Each run started with a 2 s countdown. For all speller conditions, each set of characters flashed for 135 ms, followed by an ISI of 50 ms. When subjects were instructed to copy-spell, the spelling of each letter consisted of 10 sequences without a prolonged inter-sequence interval. One sequence consists of 12 flashes. For the RC case every column and every row was flashed once. For the RASP and RASP-F cases each letter was flashed twice, however groups of letters were shuffled after each flash. Note, that for all cases the target flashed twice.

### Data analysis

We used the BBCI toolbox (http://bbci.de/toolbox) for our analysis. EEG data was band-pass filtered between 0.1 and 30 Hz with a 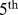 order Butterworth digital filter. In each experimental session, the data was epoched from −200 ms to 800 ms with respect to stimulus onset. Epoched EEG signals were baseline-corrected by subtracting the mean amplitudes in the −200 to 0 ms pre-stimulus interval from every epoch. Then, averaged features of the ERPs were extracted from 8 selected discriminative intervals, which were selected by a well established heuristic, which depends on signed r-values [23]. These subject-dependent intervals were located in the 100–600 ms poststimulus interval, thereby forming an averaged spatiotemporal feature vector with a dimension of 232 (i.e. 29 channels 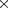
8 averaged temporal features). After that, these features from the training phase were validated with the data from the test phase with the help of a regularized linear discriminant analysis (RLDA) classifier with analytic shrinkage of the covariance matrix [23], [49]. For the evaluation of the 3 matrix spellers classification accuracies (a 0–1 loss function was used) as well as Information Transfer Rates (ITRs) were computed. ITRs are commonly used as an evaluation measurement for BCIs. The unit of ITRs is given as *bits per unit time* [bits min^−1^] and can be calculated as
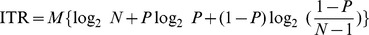
(1)


where 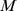 denotes the number of commands per minute and 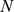 indicates the number of the possible choices in which each choice is equally probable to be selected by the user. 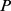 is the accuracy of the BCI (i.e. the probability that the BCI selects what the user intends). In summary ITR corresponds to the amount of information received by the system.

To further examine the effect of self-face stimuli on classification performance, we separated all self-face stimuli from non-self stimuli in the RASP-F condition, where self-faces occured in rows and non-self faces in columns. For each session and subject we performed 8-fold chronological cross-validation employing an RLDA classifier. To evaluate whether classification accuracy of self-face stimuli outperforms accuracy of non-self stimuli significantly, we performed sign-tests. The sign-test is a non-parametric test, which relies on only very few assumptions [50], [51]. Three statistical tests were performed on the cross-validated accuracies of RC vs. SF, RASP vs. SF and NSF vs. SF to test the hypothesis, whether the difference median is zero between the continuous distributions of the two random variables. Results of this test were then Bonferroni corrected [52].

Furthermore, a two way repeated measure ANOVA was performed in order to study *accuracy* and *ITR* (dependent variables) with respect to the within-subject factors *type of speller* and *number of sequences*. *Type of speller* contained three levels: RC, RASP and RASP-F. The *number of sequences* contained 10 levels (from 1 to 10).

To examine, whether ERP components were significantly different for the three types of spellers, values were first averaged across time in the following intervals with respect to stimulus onset: 130–200ms for N170 (channel 'PO7'), 280–370ms for P300 (channel 'Cz') and 400–550ms for N400f (channel ‘Cz'). The choice of intervals and electrode locations was based on previous publications in order to increase comparability of the analysis [29], [33], [43]. Then two-sample t-tests were performed with the null hypothesis of equal means. Results were then Bonferroni corrected (3 tests per ERP component were performed).

## Results

### Classification accuracy and ITR

**Figure 4:**
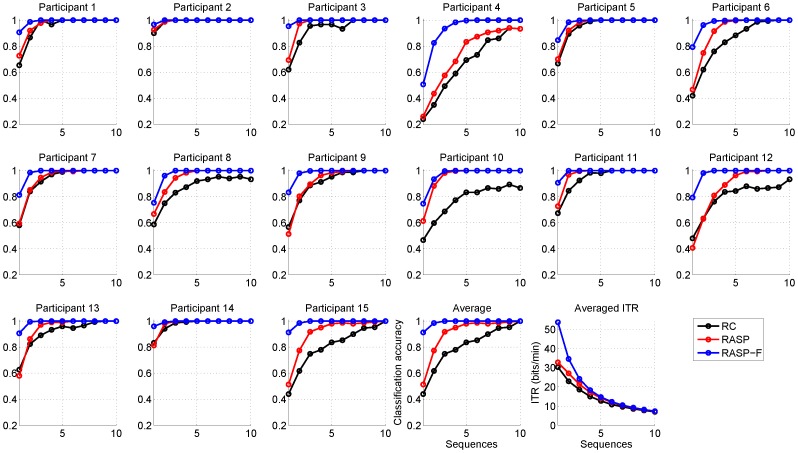
Classification accuracy curves of each subject for three conditions ('RC', 'RASP', and 'RASP-F') using one to ten sequences. Also, on the bottom right the averaged classification accuracy and ITR on all subjects are plotted.

Figure 4 depicts the classification accuracy for each subject as well as averaged accuracies and ITRs for all subjects. The number of sequences were varied from one to ten sequences (x-axis) for all three different spellers. In the RASP-F condition, on average fewer sequences (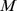  = 1.1
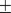
0.3) were necessary for achieving an accuracy level of 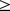
70% as compared to RC (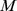  = 2.5
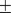
1.3) and RASP conditions (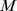  = 1.9
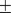
1.0). This threshold has previously been argued to be the minimum accuracy level for meaningful communication [53]. To ensure an accuracy level of 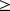
90%, the number of sequences needed were 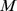 = 1.6
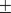
0.6, for the RASP-F condition, 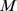  = 4.9
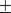
2.9 for the RC condition and 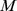  = 3.0
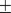
1.5 for the RASP condition.

Offline selection accuracies for selecting one symbol out of 36 by using single sequence data were 58.4%
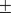
1.6% for RC, 61.3%
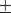
1.6% for RASP and 84.0%
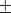
1.2% for RASP-F. In an offline analysis, we investigated classification performance and ITR as a function of the number of sequences (i.e. repetitions of the intensification). As expected, performance increased sharply with the number of repetitions.

Table 1 summarizes the results of the two-way repeated measures ANOVA. The ANOVA revealed an increase of accuracy with the number of sequences, and a difference in accuracy for the three spellers. Interaction between type of speller and number of sequences was significant. When we compared the RC and RASP conditions with single sequences, a paired t-test revealed that accuracies are not significantly different (RC 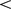 RASP, 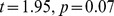). However, when increasing the number of sequences to 3, accuracies become significantly different (RC 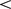 RASP, 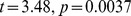). The difference between face-related stimuli and highlighted characters are significantly different for a single sequence (RC 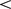 RASP-F: 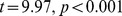; RASP 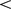 RASP-F: 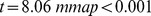) as well as for 3 sequences (RC 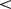 RASP-F: 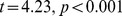; RASP 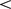 RASP-F: 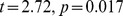).

**Table 1:** F-values and significance of the repeated measures 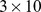 ANOVA.

		accuracy	ITR
speller	F(2,28)	54.64***	92.24***
sequences	F(9,126)	270.65***	109.02***
speller 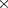 sequences	F(18,252)	14.1***	8.04***

*speller* stands for *type of speller* (RC, RASP, RASP-F) and *sequences* for *number of sequences* (1 to 10). *** corresponds to 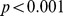

ITR among the three spellers was also significantly different. The best performance with an ITR of 53.7
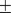
11.8 bits/min was achieved by RASP-F as compared to the 30.3
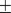
13.3 bits/min for RC and 32.8 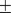 13.8 bits/min for RASP. The difference between face-related stimuli and highlighted characters was significantly enhanced for single sequence data (RC 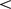 RASP-F: 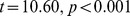; RASP 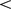 RASP-F: 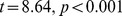) as well as for 3 repeated sequences (RC 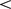 RASP-F: 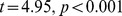; RASP 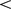 RASP-F: 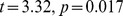).

Table 2 shows the single-trial classification accuracy [%] of the 8-fold cross-validation for each paradigm and subject. *RC* stands for Row Column, *RASP* for random set presentation, *RASP-F* for random set presentation with face stimuli, *NSF* stands for *non-self face stimuli*, while *SF* stands for *self-face stimuli*. Average performance of SF was significantly higher, when compared to any of the other methods (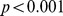, Bonferroni corrected). The stars in the table indicate the comparison of SF to NSF.

**Table 2:** Single-trial classification accuracy [%], based on 8-fold cross validation for each paradigm and subject.

Subject	RC	RASP	RASP-F	
			NSF	SF
1	93.7	88.8	86.8	90.6
2	96.1	96.6	98.0	98.6
3	90.8	89.6	91.8	94.8
4	79.1	76.5	81.6	85.2
5	89.8	89.3	88.8	91.9
6	89.2	82.8	91.7	94.3
7	88.3	86.1	81.9	89.9
8	87.4	87.3	90.1	90.4
9	90.1	82.2	91.7	97.7
10	80.1	89.0	92.8	95.2
11	89.8	93.4	96.4	97.4
12	76.0	87.1	85.8	92.6
13	85.4	87.6	95.2	95.8
14	90.8	89.9	96.9	97.9
15	79.2	79.6	91.9	93.4
Mean	87.1	87.1	90.7	93.7***

*RC* stands for Row Column, *RASP* for random set presentation, *RASP-F* for random set presentation with face stimuli, *NSF* stands for *non-self face stimuli*, while *SF* stands for *self-face stimuli*. The three stars indicates the level of significant improvement (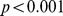) for NSF vs. SF., based on a sign test with the hypothesis of equal means.

### ERP analysis

**Figure 5:**
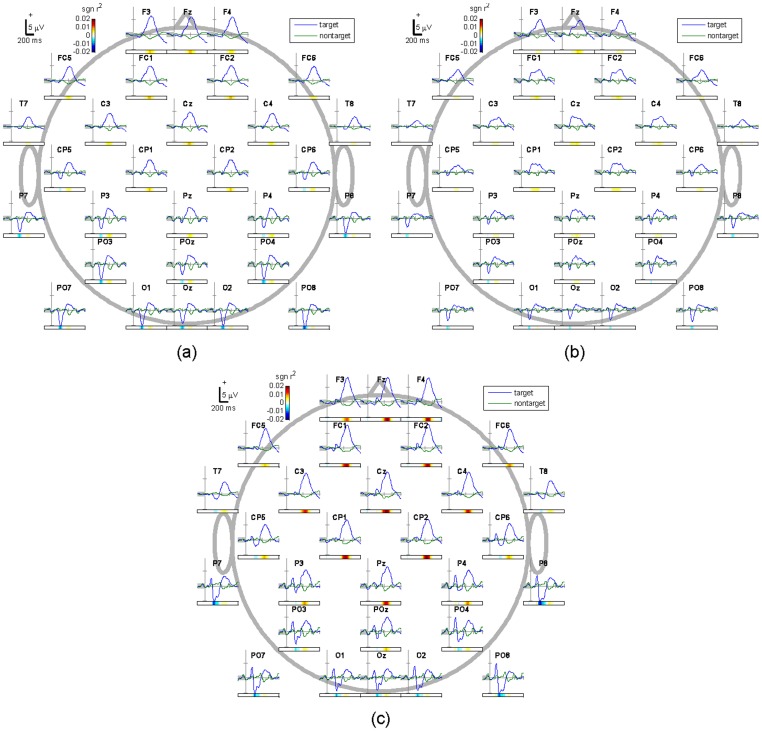
Topographic plots of grand average ERP waveform derived from the target and non-target stimuli for all 15 participants at 29 electrode channels ((a) RC, (b) RASP, (c) RASP-F). The scales of x- and y-axes for each channel are the same.

**Figure 6:**
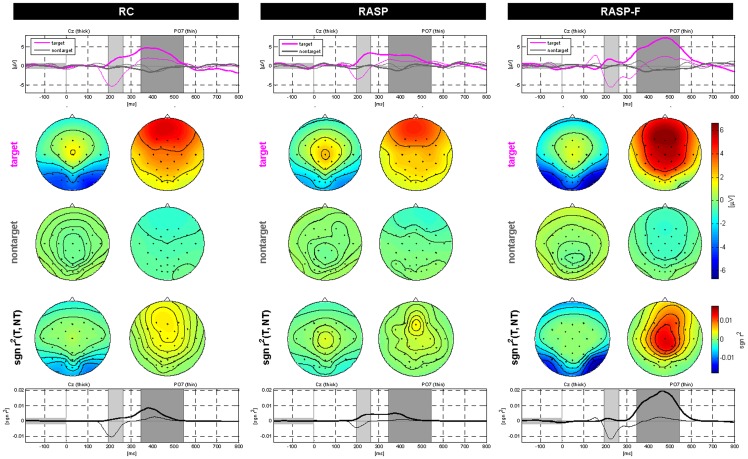
Grand average ERPs and scalp topographies for the three conditions RC, RASP, and RASP-F. Top row: ERPs for targets and nontargets at two selected electrodes Cz and PO7. The two shaded areas in each ERP plot mark the intervals for which scalp maps are shown underneath. Center: The first and second row of scalp plots indicate the ERP responses to the target and nontarget classes. Bottom row: Temporal distribution based on sgn r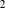 at two selected electrodes Cz and PO7. The P300 component show a higher discriminability for RASP-F as compared to the two other spellers at the central and parieto-occipital sites.

Figure 5 depicts the grand average ERP waveforms for the target and non-target stimuli for each of the three spellers. Figure 6 shows grand average ERPs and scalp topographies at representative electrodes Cz and PO7.

**Figure 7:**
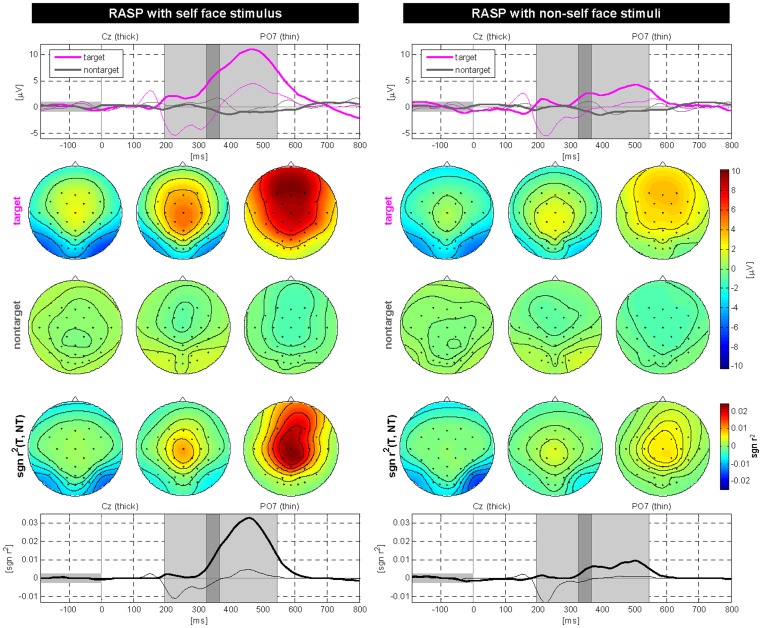
Grand average ERPs and scalp topographies for the self-face and non-self-face stimuli of the RASP-F condition. Top row: ERPs for targets and nontargets at two selected electrodes Cz and PO7. The three shaded areas extract 3 discriminative intervals. Scalp maps are shown underneath using these intervals. Center: The first and second row of scalp plots indicate the ERP responses to the target and nontarget classes. Bottom row: Temporal distribution based on sgn r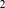 at two selected electrodes Cz and PO7. ERPs resulting from the self-face stimulus show stronger responses as those of the non-self face stimulus.

**Figure 8:**
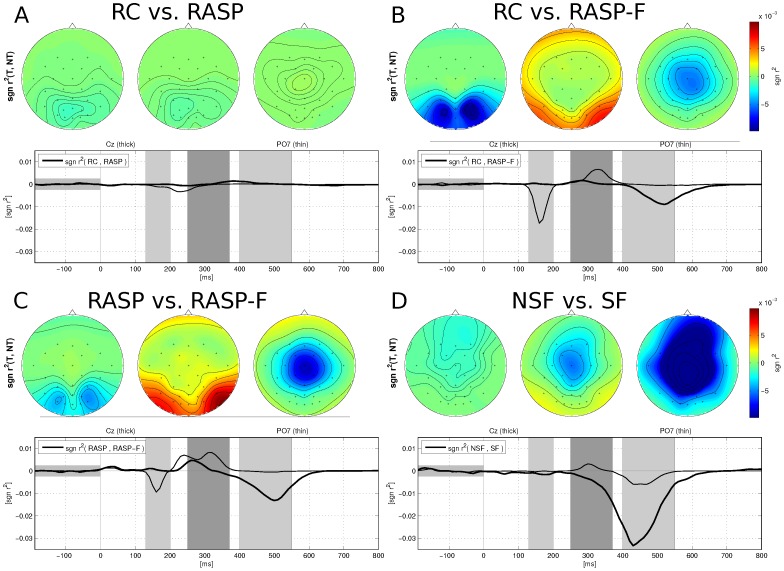
All scalp maps show 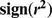
 **values, comparing** ***target stimuli*** **for the three considered paradigms (A,B,C) as well as comparing the two types of target stimuli in RASP-F: non-self-face and self-face stimuli (D).** Time courses of 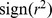 values are given below for two EEG channels (namely 'Cz' and 'PO7').

Table 3 summarizes the differences of ERP components with respect to stimulus patterns. The ANOVA revealed significant N170 and N400f amplitude differences among spellers, especially at central and parieto-occipital sites (see also center of Figure 6). As can be seen in rows 3–4, face stimuli show significantly enhanced N170 as well as N400f components when compares to RC and RASP paradigms. Furthermore, and more interestingly self-face stimuli showed stronger deflections than non-self face stimuli for P300 and N400f components (row 5; please also compare Figures 7 and 8).

**Table 3:** Examines differences of ERP components with respect to *target stimuli*.

		N170	P300	N400f
RC - RASP - RASP-F	F(2,28)	6.39**	0.32	11.04***
RC - RASP	t	−3.98**	0.92	1.75
RC - RASP-F	t	−7.31***	0.92	−5.74***
RASP - RASP-F	t	−4.82***	0.46	−6.29***
NSF - SF	t	1.49	5.01***	5.03***

First row: F-values and significance of the repeated measures one-way ANOVA. Next three rows: Show statistical significance (p-values) of ERP components having different means for the three speller conditions (two-sample t-test with the hypothesis of equal means). Last row: Shows p-values of whether ERP components have different means for the non-self face and self-face stimuli.

* - 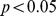 ** - 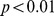 *** - 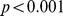

Figure 8 shows statistical differences of brain responses due to the various stimulus presentation patterns as well as stimuli. While brain responses to target stimuli were similar for RC and RASP conditions (A), face-stimuli elicited an additional N170 and a N400f component, related to face-specific processing (B and C). Self-face stimuli showed a greatly enhanced central and parietal N400f component (D).

### Error and variation on target-to-target interval analysis

**Figure 9:**
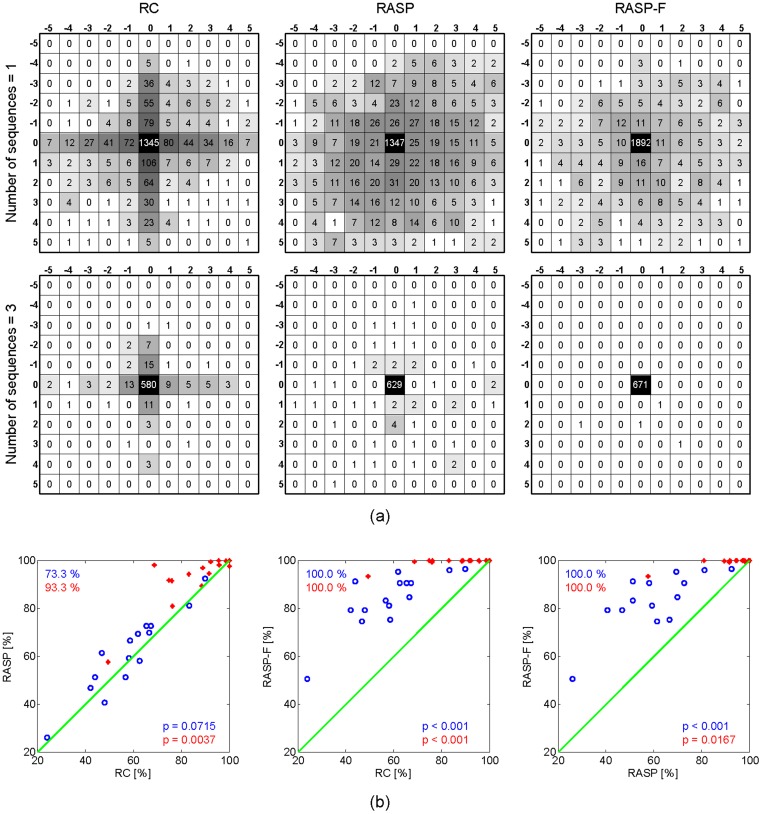
Performance comparison across spellers as number of sequences is increased. Blue circles used one repetition and red stars three repetitions. (a) Error distributions for the RC (left), RASP (center), and RASP-F (right). All target items have been centered in each matrix. The number in a black centered cell corresponds the number of correct selections and numbers in other cells represents the number of error corrected selections occurring in each cell relative to the target location for each speller. (b) Scatter plot comparing classification accuracies and significance values of various combinations of the three conditions. Each circle represents the classification accuracy of one subject.

Figure 9 illustrates the topographical distribution of errors in relation to the target item for the RC, RASP, and RASP-F based paradigms. All target items have been centered in this matrix for display purposes; the numbers in the black cells represent the number of correct selections for each paradigm. The numbers in other cells correspond to the locations of errors relative to the target location. In the RC many errors occurred in the direct neighborhood. These non-targets were flashed simultaneously with the target item in the rows and columns. The upper matrices show the results if only one sequence is considered, the lower matrices consider three sequences. As can be seen, the RASP and RASP-F based paradigms successfully reduced the number of errors, because combinations of letters were shuffled within each sequence.

**Figure 10:**
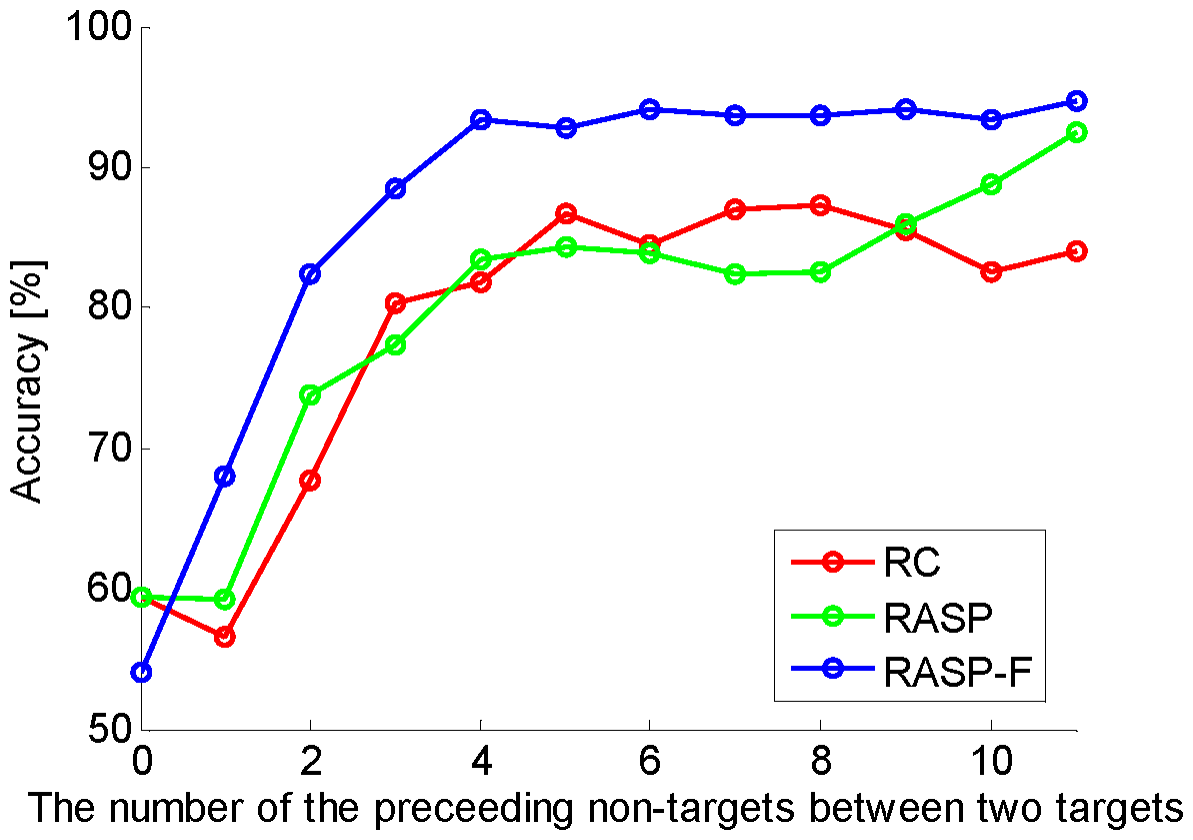
Compares the classification accuracy (y-axis) when increasing the number of non-targets preceding a target stimulus for the three conditions (x-axis). The value '0' of x-axis presents 'double flashed target'.

Additionally, we assessed the performance according to the variation of TTIs. Figure 10 depicts the performance with respect to increasing the number of the preceding non-targets between two targets. Value '0' on the 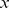-axis indicates the 'double flashed target' which occured when the same character flashed sequentially. We found that the accuracy for all considered spellers is lower when target items were frequently flashed. This effect was most prominent for less than 3 preceding non-targets. The performance gradually increased when the temporal distance between two target flashes was expanded. A minimum of four TTIs is necessary to ensure optimal performance.

## Concluding Discussion

Accurate target detection with shorter sequence data continues to be a challenging problem, since the P300 is relatively weak and usually occurs amid some ongoing background brain activities, such as spontaneous EEG as well as other task unrelated noise sources. For this reason the development of new paradigms with more effective 1) visual stimulus types, and 2) stimulus presentation patterns, which elicit stronger differential ERP responses, is considerably important for improving the performance of such BCI systems. In this study we firstly compared a recently proposed presentation method termed 'RASP' to the classic row-column P300-based paradigm and secondly compared (non-) self-face stimuli to the classical approach of simply flashing the characters. As mentioned earlier, related work for random stimulus presentation patterns have been proposed previously [33], however the insight from this previous approach was limited by the fact that two main factors were manipulated concurrently in order to avoid *adjacency-distraction errors* as well as *double flash errors*. In this study we are able to confirm previous findings, that a random set presentation approach outperforms the classical row-column paradigm [33]. By following this type of approach the *adjacency-distraction problem* can be diminished to some degree, since now most of the times the neighbouring letters do not flash simultaneously with the target letter. However, this does not eliminate the *double flash problem*. This enables us to study the effects of these two issues independently (see Figure 10). While long TTIs will increase the time for each decision and thus limit ITRs, long TTIs will at the same time increase classification accuracy. A TTI below 3 will reduce accuracy decisively, while a TTI above 4 will not increase classification accuracies enough to justify the time delay this would cause (compare Figure 10).

While the effects of face specific self-representation on brain activity has been researched extensively in the field of cognitive neuroscience [45], [54], [55], to our knowledge these findings have not been applied to enhance P300-based BCIs. In this study, we compared ERP resposes to self-face and non-self-face stimuli. Presentation of self-face stimuli produced ERPs with larger amplitudes (see Figures 5 and 8 as well as Table 3) which resulted in higher discriminability and thus lead to significantly higher classification accuracy between target and non-target characters as compared to non-self face stimuli (see Table 2). Similar findings have previously been obtained for a related setting where familiar and famous faces are compared to unfamiliar faces [40], [41].

To further increase the speed of character selection one has to focus on reducing the number of stimulus sequences used for averaging. However, usually several P300 responses must be averaged for the response to be recognized due to the low signal-to-noise ratio [56], [57]. By reshuffling and thus creating unique combinations of letters for each flash our findings indicate increased performance for the same number of sequences (see Figure 4). As can be seen from Figure 9 the performance of RASP and RASP-F increases with the number of sequences and significantly outperforms RC consistently. As can be seen from Table 1 interaction of the *type of speller* with respect to accuracy and ITR was significant, however only those subjects who performed considerably well with the RC matrix also performed well with the RASP. In those who did not, the RASP performance seemed to be visibly below that of RASP-F. Still unclear remains how the visual design of the BCI can be improved to meet peculiarities of peripheral vision such as low spatial acuity and crowding for the RASP paradigm.

Face stimuli including self- and non-self-faces yielded significantly higher accuracies and ITRs than those of highlighted characters for all participants. This implies that stimuli with higher cognitive task requirements such as facial images, are more effective than the intensified stimuli of dull characters for a P300-based BCI system. As already discussed above, previous studies have shown that faces boost BCI performance [40], [41], [43], [44]. Furthermore, familiar and famous faces have been shown to improve BCI performance even more, when compared to unknown faces [40], [41]. In this study we have analyzed these finding further by specifically comparing self-face stimuli to non-self-face stimuli; here non-self-face stimuli include unfamiliar as well as familiar faces. Thus our study can ultimately not assess the full combinatorial plentitude of stimulus types previously proposed, namely, unfamiliar vs. familiar, famous vs. familiar, famous vs. unfamiliar, self-faces vs. unfamiliar etc., rather we have chosen a particular abstraction level, i.e. self-face vs. non-self-faces (cf. also a previous study [54], which showed very prominent ERP responses, specifically to self-face stimuli).

In this study, the noticeable offline performance with an accuracy of 84.0%
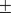
1.2% and an ITR of 53.7
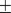
11.8 bits/min, when considering single sequences, indicates that the proposed paradigm is very promising (see Figure 4). For achieving a performance level of 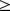
70% (described as the minimum level for communication in the literature [53]) RASP-F can reduce the overall time needed to spell a character by a factor of 2.3 on average in comparison to RC and by a factor of 1.7 in comparison to RASP.

It may be possible to further improve the performance of the proposed BCI by adopting more advanced feature extraction techniques, such as kernel PCA [58] and/or non-linear machine learning techniques, such as logistic regression or support vector machines [59]–[61].

While some individual variation is evident, the individual participants' averaged ERPs conform to the grand mean shown in Figures 5 and 6, which shows that both the target and non-target ERPs differ in several respects across spellers. N170 amplitudes were significantly enlarged at parieto-occipital sites, when face stimuli were compared to highlighted characters. P300 tends to be more pronounced at the central sites for face stimuli, against those evoked by the highlighted character (RC and RASP). Face stimuli elicited significantly higher P300s than the highlighted character (see Figure 5 and 6). This suggests higher level of cognitive components in the central areas through the face perception task. Such cognitive components associated with face perception result in more discriminative features.

We also checked the neurophysiological phenomena associated with face-specific visual self-representation in a human brain. Our findings show class-discriminative ERP patterns between self-face and non-self-face stimuli (see Figures 7 and 8). Although individual differences of ERP patterns for the face processing exist, the amplitudes of N400f for self-face stimuli were significantly larger than those for non-self-face stimuli. Besides, the N170, which is related to cognitive processing, can show large amplitudes for both self- and non-self-face stimuli.

Summarizing, a novel BCI paradigm combining random set presentation with self-face stimuli has been proposed and developed. The proposed BCI can lead to higher classification accuracy and ITRs than the conventional RC-based paradigm. The performance of the RASP-F condition yielded a single-trial classification accuracy of 84.0%
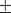
1.2% and an ITR of 53.7
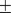
11.8 bits/min.

We would like to finally remark that our approach as virtually all other work on P300 spellers is gaze dependent. However, as pointed out in their contribution [28], [29], [62], a clear path to gaze independent BCI spellers can be pursued (see also a recent patient study contributing to the debate [63]). Future work will therefore extend the present paradigms towards gaze independency.

## References

[1] Mak JN, Wolpaw JR (2009) Clinical applications of Brain–Computer Interfaces: Current state and future prospects. Biomedical Engineering, IEEE Reviews in 2: 187–199. 10.1109/RBME.2009.2035356 PMC2862632 20442804

[2] Wolpaw J, Wolpaw EW (2012) Brain–Computer Interfaces: Principles and practice. Oxford University Press.

[3] Dornhege G, Millán JR, Hinterberger T, McFarland DJ, Müller KR (2007) Toward Brain–Computer Interfacing. MIT press.

[4] Wolpaw JR, Birbaumer N, McFarland DJ, Pfurtscheller G, Vaughan TM (2002) Brain–Computer Interfaces for communication and control. Clinical Neurophysiology 113: 767–791. 12048038 10.1016/s1388-2457(02)00057-3

[5] Kübler A, Kotchoubey B, Kaiser J, Wolpaw JR, Birbaumer N (2001) Brain–Computer communication: Unlocking the locked in. Psychological Bulletin 127: 358. 11393301 10.1037/0033-2909.127.3.358

[6] Nijboer F, Sellers E, Mellinger J, Jordan M, Matuz T, et al (2008) A P300-based Brain–Computer Interface for people with amyotrophic lateral sclerosis. Clinical Neurophysiology 119: 1909–1916. 18571984 10.1016/j.clinph.2008.03.034 PMC2853977

[7] Farwell LA, Donchin E (1988) Talking off the top of your head: Toward a mental prosthesis utilizing event-related brain potentials. Electroencephalography and Clinical Neurophysiology 70: 510–523. 2461285 10.1016/0013-4694(88)90149-6

[8] Gao S, Wang Y, Gao X, Hong B (2014) Visual and auditory Brain–Computer Interfaces. Biomedical Engineering, IEEE Transactions on 61: 1436–1447. 10.1109/TBME.2014.2300164 24759277

[9] Krusienski DJ, Sellers EW, Cabestaing F, Bayoudh S, McFarland DJ, et al (2006) A comparison of classification techniques for the P300 speller. Journal of Neural Engineering 3: 299. 17124334 10.1088/1741-2560/3/4/007

[10] Sellers EW, Krusienski DJ, McFarland DJ, Vaughan TM, Wolpaw JR (2006) A P300 Event-Related Potential Brain–Computer Interface (BCI): The effects of matrix size and inter stimulus interval on performance. Biological Psychology 73: 242–252. 16860920 10.1016/j.biopsycho.2006.04.007

[11] Gibert G, Attina V, Mattout J, Maby E, Bertrand O (2008) Size enhancement coupled with intensification of symbols improves P300 speller accuracy. In: 4th BCI Workshop and Training Course.

[12] Martens S, Hill N, Farquhar J, Schölkopf B (2009) Overlap and refractory effects in a Brain–Computer Interface speller based on the visual P300 Event-Related Potential. Journal of Neural Engineering 6: 026003. 19255462 10.1088/1741-2560/6/2/026003

[13] Takano K, Komatsu T, Hata N, Nakajima Y, Kansaku K (2009) Visual stimuli for the P300 Brain–Computer Interface: A comparison of white/gray and green/blue flicker matrices. Clinical Neurophysiology 120: 1562–1566. 19560965 10.1016/j.clinph.2009.06.002

[14] Salvaris M, Sepulveda F (2009) Visual modifications on the P300 speller BCI paradigm. Journal of Neural Engineering 6: 046011. 19602731 10.1088/1741-2560/6/4/046011

[15] Liu T, Goldberg L, Gao S, Hong B (2010) An online Brain–Computer Interface using non-flashing visual evoked potentials. Journal of Neural Engineering 7: 036003. 20404396 10.1088/1741-2560/7/3/036003

[16] McFarland DJ, Sarnacki WA, Townsend G, Vaughan T, Wolpaw JR (2011) The P300-based brain–computer interface (BCI): Effects of stimulus rate. Clinical Neurophysiology 122: 731–737. 21067970 10.1016/j.clinph.2010.10.029 PMC3050994

[17] Allison BZ, Pineda JA (2006) Effects of SOA and flash pattern manipulations on ERPs, performance, and preference: Implications for a BCI system. International Journal of Psychophysiology 59: 127–140. 16054256 10.1016/j.ijpsycho.2005.02.007

[18] Jin J, Sellers EW, Wang X (2012) Targeting an efficient target-to-target interval for P300 speller Brain–Computer Interfaces. Medical & Biological Engineering & Computing 50: 289–296. 22350331 10.1007/s11517-012-0868-x PMC3646326

[19] Polprasert C, Kukieattikool P, Demeechai T, Ritcey JA, Siwamogsatham S (2013) New stimulation pattern design to improve P300-based matrix speller performance at high flash rate. Journal of Neural Engineering 10: 036012. 23612883 10.1088/1741-2560/10/3/036012

[20] Xu N, Gao X, Hong B, Miao X, Gao S, et al (2004) BCI competition 2003-data set IIb: Enhancing P300 wave detection using ICA-based subspace projections for BCI applications. Biomedical Engineering, IEEE Transactions on 51: 1067–1072. 10.1109/TBME.2004.826699 15188880

[21] Serby H, Yom-Tov E, Inbar GF (2005) An improved P300-based Brain-Computer Interface. Neural Systems and Rehabilitation Engineering, IEEE Transactions on 13: 89–98. 10.1109/TNSRE.2004.841878 15813410

[22] Rivet B, Souloumiac A, Attina V, Gibert G (2009) xDAWN algorithm to enhance evoked potentials: application to Brain–Computer Interface. Biomedical Engineering, IEEE Transactions on 56: 2035–2043. 10.1109/TBME.2009.2012869 19174332

[23] Blankertz B, Lemm S, Treder M, Haufe S, Müller KR (2011) Single-trial analysis and classification of ERP components–A tutorial. NeuroImage 56: 814–825. 20600976 10.1016/j.neuroimage.2010.06.048

[24] Krusienski DJ, Sellers EW, McFarland DJ, Vaughan TM, Wolpaw JR (2008) Toward enhanced P300 speller performance. Journal of Neuroscience Methods 167: 15–21. 17822777 10.1016/j.jneumeth.2007.07.017 PMC2349091

[25] Rakotomamonjy A, Guigue V (2008) BCI competition III: Dataset II-ensemble of SVMs for BCI P300 speller. Biomedical Engineering, IEEE Transactions on 55: 1147–1154. 10.1109/TBME.2008.915728 18334407

[26] Guger C, Daban S, Sellers E, Holzner C, Krausz G, et al (2009) How many people are able to control a P300-based Brain–Computer Interface (BCI)? Neuroscience Letters 462: 94–98. 19545601 10.1016/j.neulet.2009.06.045

[27] Fazel-Rezai R, Abhari K (2009) A region-based P300 speller for Brain–Computer Interface. Electrical and Computer Engineering, Canadian Journal of 34: 81–85.

[28] Treder MS, Blankertz B (2010) (C)overt attention and visual speller design in an ERP-based Brain–Computer Interface. Behavioral and Brain Functions 6: 28. 20509913 10.1186/1744-9081-6-28 PMC2904265

[29] Treder MS, Schmidt NM, Blankertz B (2011) Gaze-independent Brain–Computer Interfaces based on covert attention and feature attention. Journal of Neural Engineering 8: 066003. 21975312 10.1088/1741-2560/8/6/066003

[30] Kindermans PJ, Verschore H, Verstraeten D, Schrauwen B (2012) A P300 BCI for the masses: Prior information enables instant unsupervised spelling. In: Advances In Neural Information Processing Systems 25.

[31] Kindermans PJ, Tangermann M, Müller KR, Schrauwen B (2014) Integrating dynamic stopping, transfer learning and language models in an adaptive zero-training ERP speller. Journal of Neural Engineering 11: 035005. 24834896 10.1088/1741-2560/11/3/035005

[32] Spüler M, Rosenstiel W, Bogdan M (2012) Online adaptation of a c-VEP Brain–Computer Interface (BCI) based on Error-related potentials and unsupervised learning. PloS One 7: e51077. 23236433 10.1371/journal.pone.0051077 PMC3517594

[33] Townsend G, LaPallo B, Boulay C, Krusienski D, Frye G, et al (2010) A novel P300-based Brain–Computer Interface stimulus presentation paradigm: Moving beyond rows and columns. Clinical Neurophysiology 121: 1109–1120. 20347387 10.1016/j.clinph.2010.01.030 PMC2879474

[34] Townsend G, Shanahan J, Ryan DB, Sellers EW (2012) A general P300 Brain–Computer Interface presentation paradigm based on performance guided constraints. Neuroscience Letters 531: 63–68. 22960261 10.1016/j.neulet.2012.08.041 PMC3646331

[35] Kanwisher NG (1987) Repetition blindness: Type recognition without token individuation. Cognition 27: 117–143. 3691023 10.1016/0010-0277(87)90016-3

[36] Sanders A, Lamers J (2002) The Eriksen flanker effect revisited. Acta Psychologica 109: 41–56. 11766139 10.1016/s0001-6918(01)00048-8

[37] Jin J, Allison BZ, Sellers EW, Brunner C, Horki P, et al (2011) Optimized stimulus presentation patterns for an Event-Related Potential EEG-based Brain–Computer Interface. Medical & Biological Engineering & Computing 49: 181–191. 20890671 10.1007/s11517-010-0689-8

[38] Jin J, Allison BZ, Sellers EW, Brunner C, Horki P, et al (2011) An adaptive P300-based control system. Journal of Neural Engineering 8: 036006. 21474877 10.1088/1741-2560/8/3/036006 PMC4429775

[39] Bentin S, Allison T, Puce A, Perez E, McCarthy G (1996) Electrophysiological studies of face perception in humans. Journal of Cognitive Neuroscience 8: 551–565. 20740065 10.1162/jocn.1996.8.6.551 PMC2927138

[40] Kaufmann T, Schulz S, Grünzinger C, Kübler A (2011) Flashing characters with famous faces improves ERP-based Brain–Computer Interface performance. Journal of Neural Engineering 8: 056016. 21934188 10.1088/1741-2560/8/5/056016

[41] Kaufmann T, Schulz SM, Köblitz A, Renner G, Wessig C, et al (2013) Face stimuli effectively prevent Brain–Computer Interface inefficiency in patients with neurodegenerative disease. Clinical Neurophysiology 124: 893–900. 23246415 10.1016/j.clinph.2012.11.006

[42] Blankertz B, Sannelli C, Halder S, Hammer EM, Kübler A, et al (2010) Neurophysiological predictor of SMR-based BCI performance. Neuroimage 51: 1303–1309. 20303409 10.1016/j.neuroimage.2010.03.022

[43] Zhang Y, Zhao Q, Jin J, Wang X, Cichocki A (2012) A novel BCI based on ERP components sensitive to configural processing of human faces. Journal of Neural Engineering 9: 026018. 22414683 10.1088/1741-2560/9/2/026018

[44] Jin J, Allison BZ, Kaufmann T, Kübler A, Zhang Y, et al (2012) The changing face of P300 BCIs: A comparison of stimulus changes in a P300 BCI involving faces, emotion, and movement. PloS One 7: e49688. 23189154 10.1371/journal.pone.0049688 PMC3506655

[45] Miyakoshi M, Kanayama N, Iidaka T, Ohira H (2010) EEG evidence of face-specific visual selfrepresentation. NeuroImage 50: 1666–1675. 20079852 10.1016/j.neuroimage.2010.01.030

[46] Ninomiya H, Onitsuka T, Chen CH, Sato E, Tashiro N (1998) P300 in response to the subject's own face. Psychiatry and Clinical Neurosciences 52: 519–522. 10215014 10.1046/j.1440-1819.1998.00445.x

[47] Yeom SK, Suk HI, Lee SW (2013) Person authentication from neural activity of face-specific visual self-representation. Pattern Recognition 46: 1159–1169.

[48] Wintink AJ, Segalowitz SJ, Cudmore LJ (2001) Task complexity and habituation effects on frontal P300 topography. Brain and Cognition 46: 307–311. 11527356 10.1016/s0278-2626(01)80090-7

[49] Lemm S, Blankertz B, Dickhaus T, Müller KR (2011) Introduction to machine learning for brain imaging. NeuroImage 56: 387–399. 21172442 10.1016/j.neuroimage.2010.11.004

[50] Wilcoxon F (1945) Individual comparisons by ranking methods. Biometrics 1: 80–83.

[51] Siegel S (1956) Nonparametric statistics for the behavioral sciences. McGraw-hill

[52] Bonferroni CE (1936) Teoria statistica delle classi e calcolo delle probabilita. Libreria internazionale Seeber

[53] Kübler A, Birbaumer N (2008) Brain–Computer Interfaces and communication in paralysis: Extinction of goal directed thinking in completely paralysed patients? Clinical Neurophysiology 119: 2658–2666. 18824406 10.1016/j.clinph.2008.06.019 PMC2644824

[54] Keyes H, Brady N, Reilly RB, Foxe JJ (2010) My face or yours? Event-Related Potential correlates of self-face processing. Brain and Cognition 72: 244–254. 19854553 10.1016/j.bandc.2009.09.006

[55] Uddin LQ, Kaplan JT, Molnar-Szakacs I, Zaidel E, Iacoboni M (2005) Self-face recognition activates a frontoparietal “mirror” network in the right hemisphere: An event-related fMRI study. NeuroImage 25: 926–935. 15808992 10.1016/j.neuroimage.2004.12.018

[56] Polich J (2007) Updating P300: An integrative theory of P3a and P3b. Clinical Neurophysiology 118: 2128–2148. 17573239 10.1016/j.clinph.2007.04.019 PMC2715154

[57] Pritchard WS (1981) Psychophysiology of P300. Psychological Bulletin 89: 506. 7255627

[58] Schölkopf B, Smola A, Müller KR (1998) Nonlinear component analysis as a kernel eigenvalue problem. Neural Computation 10: 1299–1319.

[59] Müller KR, Anderson CW, Birch GE (2003) Linear and nonlinear methods for Brain–Computer Interfaces. Neural Systems and Rehabilitation Engineering, IEEE Transactions on 11: 165–169. 10.1109/TNSRE.2003.814484 12899264

[60] Bishop CM (2006) Pattern Recognition and Machine Learning, volume 1. springer.

[61] Vapnik V (1995) The Nature of Statistical Learning Theory. Springer.

[62] Riccio A, Mattia D, Simione L, Olivetti M, Cincotti F (2012) Eye-gaze independent EEG-based Brain–Computer Interfaces for communication. Journal of Neural Engineering 9: 045001. 22831893 10.1088/1741-2560/9/4/045001

[63] Kaufmann T, Holz EM, Kübler A (2013) Comparison of tactile, auditory, and visual modality for brain-computer interface use: a case study with a patient in the locked-in state. Frontiers in neuroscience 7 10.3389/fnins.2013.00129 PMC3721006 23898236

